# Improvements in ECG accuracy for diagnosis of left ventricular hypertrophy in obesity

**DOI:** 10.1136/heartjnl-2015-309201

**Published:** 2016-08-02

**Authors:** Oliver J Rider, Ntobeko Ntusi, Sacha C Bull, Richard Nethononda, Vanessa Ferreira, Cameron J Holloway, David Holdsworth, Masliza Mahmod, Jennifer J Rayner, Rajarshi Banerjee, Saul Myerson, Hugh Watkins, Stefan Neubauer

**Affiliations:** 1 Radcliffe Department of Medicine, Division of Cardiovascular Medicine and University of Oxford Centre for Clinical Magnetic Resonance Research, University of Oxford, Oxford, UK; 2 Division of Cardiology, Department of Medicine Research, University of Capetown, South Africa; 3 Chris Hani Baragwanath Hospital, Soweto & University of Witwatersrand, Johannesburg, South Africa; 4 St Vincent's Hospital, Sydney, Australia

## Abstract

**Objectives:**

The electrocardiogram (ECG) is the most commonly used tool to screen for left ventricular hypertrophy (LVH), and yet current diagnostic criteria are insensitive in modern increasingly overweight society. We propose a simple adjustment to improve diagnostic accuracy in different body weights and improve the sensitivity of this universally available technique.

**Methods:**

Overall, 1295 participants were included—821 with a wide range of body mass index (BMI 17.1–53.3 kg/m^2^) initially underwent cardiac magnetic resonance evaluation of anatomical left ventricular (LV) axis, LV mass and 12-lead surface ECG in order to generate an adjustment factor applied to the Sokolow–Lyon criteria. This factor was then validated in a second cohort (n=520, BMI 15.9–63.2 kg/m^2^).

**Results:**

When matched for LV mass, the combination of leftward anatomical axis deviation and increased BMI resulted in a reduction of the Sokolow–Lyon index, by 4 mm in overweight and 8 mm in obesity. After adjusting for this in the initial cohort, the sensitivity of the Sokolow–Lyon index increased (overweight: 12.8% to 30.8%, obese: 3.1% to 27.2%) approaching that seen in normal weight (37.8%). Similar results were achieved in the validation cohort (specificity increased in overweight: 8.3% to 39.1%, obese: 9.4% to 25.0%) again approaching normal weight (39.0%). Importantly, specificity remained excellent (>93.1%).

**Conclusions:**

Adjusting the Sokolow–Lyon index for BMI (overweight +4 mm, obesity +8 mm) improves the diagnostic accuracy for detecting LVH. As the ECG, worldwide, remains the most widely used screening tool for LVH, implementing these findings should translate into significant clinical benefit.

## Introduction

As electrocardiographic determination of left ventricular hypertrophy (LVH) is not costly, is simple to perform and yields indices linked to mortality,^1^ ^2^ it is widely used in clinical practice and appears in international guidelines.^3^ ^4^ As a result, the electrocardiogram (ECG) remains, worldwide, the most commonly used screening tool.

The two most commonly used ECG criteria are the Sokolow–Lyon index^5^ and the Cornell criteria. Despite their routine clinical use, the diagnostic accuracy of these surface ECG criteria is limited, with poor sensitivity for the detection of LVH.^6^ ^7^ Indeed, the original authors of the Sokolow–Lyon index report only a 32% sensitivity, making its uptake into clinical practice and its continued use surprising. Obesity, which is now pandemic in the Western world, results in three distinct processes that affect the surface ECG—lateral displacement of the anatomical left ventricular (LV) axis, increased chest wall fat and increased pericardial fat mass, all of which decrease voltage amplitude on the ECG.^8–10^ This reduces the diagnostic sensitivity of the surface ECG even further and is likely to render ECG criteria generated in a society with a much lower body mass index (BMI) (∼20 kg/m^2^ in 1949)^11^ to be obsolete in the modern age of obesity, where LVH is increasingly seen.^12^

Although previous attempts have been made at improving the diagnostic accuracy of the ECG by adjusting for body habitus, they have either relied on echocardiographic determination of LV mass,^13^ ^14^ which is severely limited in obesity, or have not taken into account the anatomical LV axis deviation that accompanies obesity.^15^ Furthermore, although all report diagnostic performance improvement, these studies all implemented complex statistical modelling to derive adjustment formula that could be applied to the ECG to adjust for obesity.^6^ Hence, they are not practical to perform as a quick screening tool and have failed to enter routine clinical practice.

The aim of this study was to develop a simple BMI-based adjustment factor for the ECG that could be used in everyday clinical practice to improve diagnostic accuracy of the detection of LVH in the modern population, where obesity is increasingly prevalent.

## Methods

### Initial cohort population

**Figure 1:**
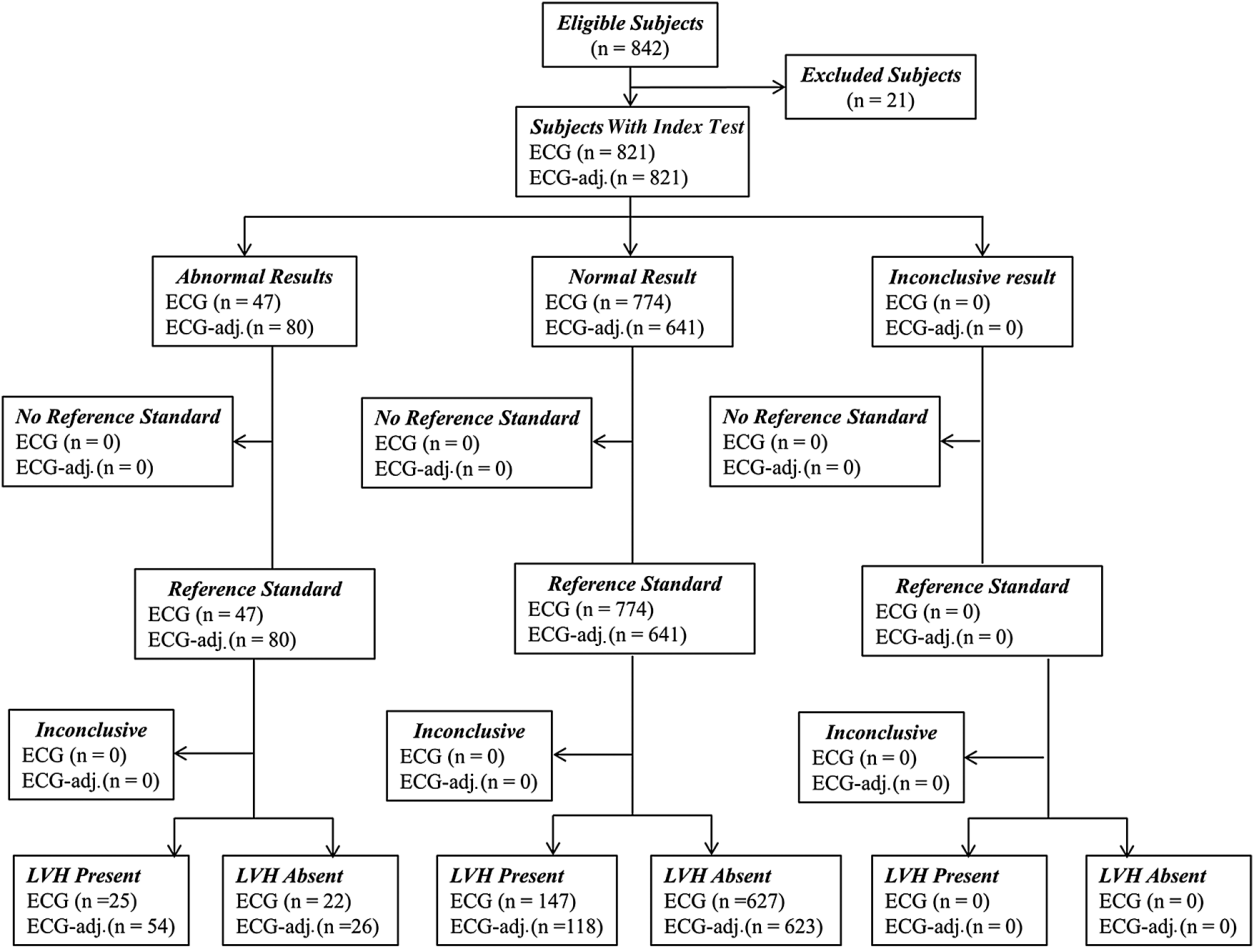
Flow chart of subjects through the study. ECG, electrocardiogram; LVH, left ventricular hypertrophy.

All research data acquisition was approved by the local research ethics committee and informed written consent was obtained from each participant. Participants were recruited from the Oxfordshire population to studies within the Oxford Centre for Clinical Magnetic Resonance Research (OCMR) between 2005 and 2012. A flow chart of subjects through phase I of the study is shown in figure 1. All participants were subject to the same exclusion criteria—pregnancy, under 18 years of age, claustrophobia and metallic foreign body. Due to effects on ECG voltage amplitude, subjects with complete left or right bundle branch block (LBBB or RBBB), chronic obstructive pulmonary disease, imaging evidence of myocardial infarction, hypertrophic cardiomyopathy or significant (>1 cm) pericardial effusion were excluded. Of the identified 842 subjects fulfilling inclusion criteria, 21 were excluded from final analysis (15 with either LBBB or RBBB, 4 without Half Fourier Acquisition Single shot Turbo spin Echo (HASTE) imaging and 2 with non-diagnostic cardiovascular magnetic resonance (CMR) quality). A retrospective analysis of the ECGs and CMR scans was performed on the remaining 821 participants (♂n=450, ♀n=371, aged 19–87 years) across a wide range of BMI (17.1–53.3 kg/m^2^). The group was predominantly Caucasian in origin.

### Validation cohort

All 520 adult participants for the validation cohort were recruited in the same fashion, with additional recruitment from the Division of Cardiology, Department of Medicine Research, University of Capetown, South Africa. To maximise generalisability of the study, within this cohort, 9.3% were African (normal weight 60%, overweight 19%, obese 21%) and 90.7% were Caucasian. Inclusion and exclusion criteria were as described above. A retrospective analysis was performed on these 520 adult participants (♂n=228, BMI 15.9–63.2 kg/m^2^).

### Anthropometric data

Height and weight were measured using a digital station (Seca, UK) and used to calculate BMI. Subjects were grouped according to World Health Organisation BMI categories: normal (18.5–24.9 kg/m^2^), overweight (25.0–29.9 kg/m^2^) or obese (>30.0 kg/m^2^). Blood pressure was recorded (DINAMAP-1846-SX, Critikon Corp.) (table 1).

**Table 1:** The relationship between body mass index (BMI), electrocardiogram left ventricular hypertrophy criteria, CMR left ventricular (LV) mass and anatomical LV axis in the initial and validation cohorts

	Normal weight	Overweight	Obese
Initial cohort (n=821)	n=263	n=296	n=262
Age (years)	50 (15)	55 (15)	54 (14)
Height (cm)	171 (9)	171 (10)	168 (9)
Systolic blood pressure (mm Hg)	126 (17)	136 (19)	135 (19)
Diastolic blood pressure (mm Hg)	75 (9)	80 (10)	81 (10)
BMI (kg/m^2^)	23 (2)	27 (1)	35 (6)
Frontal plane axis (°)	45 (12)	38 (13)	28 (13)
Sagittal plane axis (°)	137 (12)	147 (12)	154 (11)
LV mass (g)	119 (41)	137 (46)	141 (42)
Sokolow–Lyon voltage (mm)	23 (8)	21 (7)	19 (6)
Cornell voltage (mm)	13 (7)	14 (8)	14 (6)

	Normal weight	Overweight	Obese
Validation cohort (n=520)	n=206	n=169	n=145
Age (years)	52 (13)	52 (11)	54 (11)
Height (cm)	170 (9)	172 (9)	171 (10)
BMI (kg/m^2^)	22 (2)	27 (1)	34 (4)
LV mass (g)	100 (31)	113 (39)	121 (40)
Sokolow–Lyon voltage (mm)	21 (8)	19 (9)	17 (6)

## Magnetic resonance imaging

### LV axis

**Figure 2:**
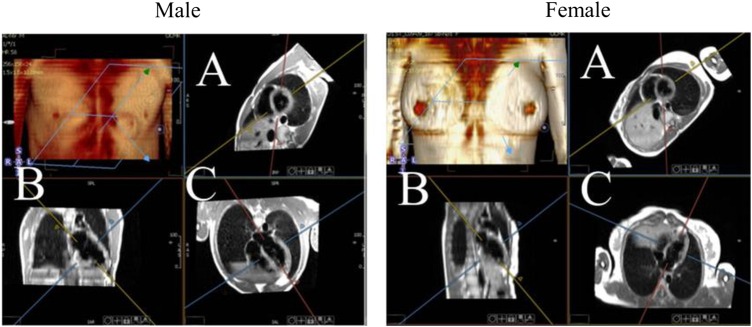
Defining the coronal anatomical left ventricular axis using multiplanar reconstruction. Images show the coronal axis in the (A) short axis, (B) vertical long axis and (C) horizontal axis views.

The anatomical axis of the LV was determined in the coronal and sagittal planes using multiplanar reconstruction of transverse thoracic HASTE images within cmr42 (Circle Cardiovascular Imaging, Calgary, Canada). The anatomical LV axis was defined as the plane between the centre of the mitral valve and the LV apex determined on both the horizontal and vertical long axis views. The LV axis in both planes (degrees 0° horizontal) was determined using a commercially available screen protractor (Screen Protractor 4.0, Iconico, New York, USA). Representative images of this process are shown in figure 2.

### LV mass and analysis

All imaging was ECG gated and acquired during breath-hold. In brief, a short axis stack of LV images was acquired (slice thickness 7 mm, gap 3 mm) using a steady-state free precession sequence (echo time of 1.5 ms, repetition time of 3.0 ms, temporal resolution of 47.84 ms, flip angle of 60°) as previously described.^16–18^ Image analysis for LV mass was performed using cmr42 by a single experienced operator with >9 years of CMR experience (OJR), as previously described.^19^ LVH was defined as >2SD higher than the published mean of the Oxfordshire population from which the study sample was taken (>165 g in men, >150 g in women).^20^ On repeat analysis of 20 scans, intraobserver variability was determined to be 6.1%. This is in keeping with previous reports.^20^

### ECG recording and analysis

A standard 12-lead ECG was performed in all participants (Fukuda Denshi Systems, UK) on the same day as the CMR. ECG measurements were performed manually by two experienced readers with >10 years of experience in ECG interpretation (OJR & DH). The analysis was blinded to participant's BMI and LV mass. The following ECG criteria were examined as reference standards: Sokolow–Lyon voltage (S-wave V_1_+R-wave lead V_5_ or V_6_)^5^ and Cornell voltage (R-wave aVL+S-wave V_3_).^21^ Measurements were taken to the nearest 0.1 mV. On repeat analysis (25 ECGs), interobserver variability for ECG analysis was <0.1 mV.

### Statistical analysis

All statistics were analysed using commercial software (SPSS V.20, Chicago, Illinois, USA). All data were normally distributed according to Kolmogorov-Smirnov testing and the results are presented as mean (SD). Group comparison data were analysed using ANOVA with Bonferroni correction. Analysis of the effects of BMI and LV axis on ECG criteria was performed using Analysis of Covariance (ANCOVA) with post hoc Bonferroni correction. Homogeneity of variance was tested with Levene's tests and visually with residual plots. Sensitivity and values were calculated before and after correction for BMI and presented with 95% confidence intervals (CIs). Positive and negative predictive values and diagnostic accuracy are also calculated. To compare the diagnostic accuracy improvements, χ^2^ and McNemar testing were performed. Data are presented as receiver operating characteristic (ROC) curves and overall diagnostic accuracy was interrogated using the Youden index. The values of p<0.05 were considered as statistically significant.

## Results

### CMR-derived and ECG-derived LVH for the initial study cohort

Of the 821 participants, 172 had CMR-defined LVH (21.0%). Despite an increase in LV mass with obesity (22 g, p<0.001), Sokolow–Lyon index voltage criteria decreased by 4 mm (p<0.001) and Cornell criteria did not increase (p>0.99, table 1). This suggests that both criteria are significantly affected by obesity and that the Sokolow–Lyon would be more suitable for a BMI adjustment factor.

### The effect of obesity on diagnostic accuracy of ECG criteria

**Figure 3:**
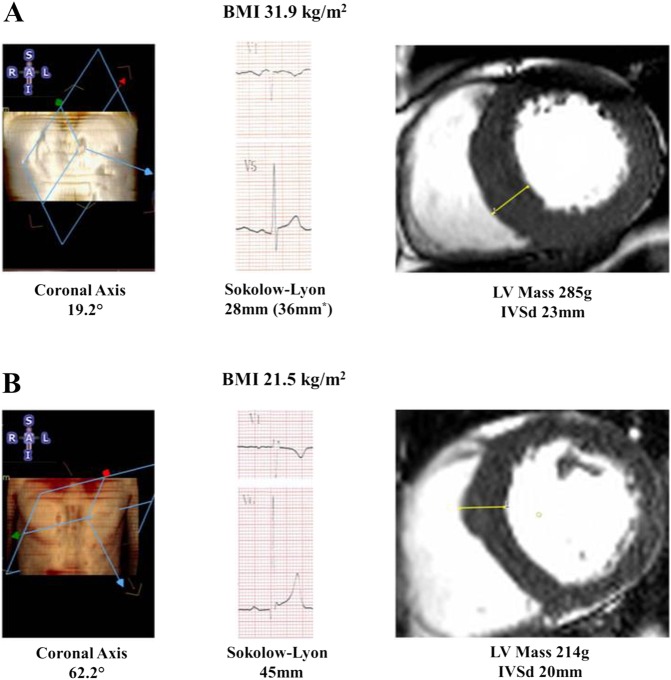
The effect of obesity and leftward axis deviation on electrocardiogram (ECG) voltage criteria. (A) Clear left ventricular hypertrophy (LVH) in obesity with leftward axis and negative Sokolow–Lyon criteria for LVH, which becomes positive only when adjusted for body mass index (BMI) (by +8 mm) and (B) clear LVH in a normal weight participant with normal left ventricular (LV) anatomical axis and positive Sokolow–Lyon criteria for LVH. IVSd, Intraventricular Septum in Diastole.

The Sokolow–Lyon criteria had poor sensitivity of 14.7% (CI 10.0% to 20.8%) but excellent specificity of 96.7% (95.1% to 97.8%) for the detection of CMR-determined LVH. Overall, diagnostic accuracy was very poor; ROC area under curve (AUC) was 0.55, Youden index was 0.11 (CI 0.05 to 0.16), positive predictive value was 0.52 (CI 0.37 to 0.67) and negative predictive value was 0.81 (CI 0.78 to 0.83) (figure 3).

However, this diagnostic performance was also too dependent on body habitus. The diagnostic sensitivity of the Sokolow–Lyon index is reduced with increasing BMI (normal 37.8% (CI 24.1% to 53.9%), overweight 12.8% (CI 6.9% to 22.7%) and obesity 3.1% (CI 0.4% to 10.8%), χ^2^ 12.6, p=0.02). In contrast, the specificity was seen to increase with increasing obesity (normal weight 92.9% (88.8% to 95.6%), overweight 98.2% (95.5% to 99.3%) and obesity 99.0% (96.4% to 99.9%), table 2). As a result, in obesity, where the decrease in voltage amplitude greatly limits the ability to generate a positive result, the Sokolov–Lyon criterion performs extremely poorly (ROC AUC 0.51, Youden index 0.02 (CI −0.01 to 0.06)).

**Table 2:** Diagnostic accuracy of unadjusted and body mass index-adjusted electrocardiogram criteria for anatomic

	Sensitivity (%)	Specificity (%)	Positive predictive value (%)	Negative predictive valve (%)	Diagnostic accuracy (%)
Initial cohort (n=821), Sokolow–Lyon index >35 mm (*=p<0.05 for adjusted vs non-adjusted)					
Total					
Unadjusted	14·7 (10·0 to 20·8)	96·7 (95·1 to 97·8)	53.2 (38.1 to 67.9)	81.4 (78.5 to 84.1)	79.8
Adjusted	31·4 (24·9 to 38·7)*	94·7 (92·7 to 96·1)*	66.25 (54.8 to 76.5)	84.4 (81.4 to 86.7)	82.6
Normal weight					
Unadjusted	37·8 (24·1 to 53·9)	92·9 (88·8 to 95·6)	46.67 (28.3 to 65.7)	97.1 (95.7 to 98.2)	85.5
Overweight					
Unadjusted	12·8 (6·9 to 22·7)	98·2 (95·5 to 99·3)	69.2 (38.6 to 90.9)	78.6 (73.4 to 83.2)	78.2
Adjusted	30·8 (20·2 to 43·3)*	96·9 (93·8 to 98·8)*	75.0 (55.1 to 89.3)	82.5 (77.4 to 86.6)	81.8
Obese					
Unadjusted	3·1 (0·4 to 10.8)	99·0 (96·4 to 99·9)	50.0 (6.76 to 93.24)	76.0 (70.3 to 81.1)	75.3
Adjusted	27·2 (18·0 to 39·0)*	97·9 (94·8 to 99·2)*	80.9 (58.1 to 94.6)	81.1 (75.5 to 85.9)	81.4
Validation cohort (n=520), Sokolow–Lyon index >35 mm (*=p<0.05 for adjusted vs non-adjusted)					
Unadjusted	17·9 (8·9 to 28·7)	97·6 (95·7 to 98·8)	50.0 (28.2 to 71.8)	89.4 (86.3 to 91.9)	87.7
Adjusted	28.1 (17·6 to 40·8)*	95.2 (92·8 to 96·9)*	45.0 (29.3 to 61.6)	90.4 (87.4 to 92.3)	86.9
Normal weight					
Unadjusted	39·0 (13·9 to 68·2)	95·4 (91·5 to 97·8)	35.7 (12.8 to 64.9)	95.9 (92.1 to 98.2)	91.9
Overweight					
Unadjusted	8·3 (1·1 to 28·0)	98·6 (95·1 to 99·8)	50.1 (6.8 to 93.0)	87.1 (81.0 to 91.9)	86.2
Adjusted	39·1 (19·7 to 61·5)*	93·1 (87·6 to 96·6)*	47.4 (24.5 to 71.1)	90.1 (84.5 to 94.7)	85.6
Obese					
Unadjusted	9.4 (1·9 to 25·0)	99·1 (95·0 to 99·9)	75.0 (19.4 to 99.4)	79.0 (71.2 to 85.5)	78.6
Adjusted	25·0 (11.5 to 43·4)*	97·3 (92·2 to 99·4)*	72.7 (39.0 to 93.9)	81.5 (73.8 to 87.8)	81.3

The Cornell criteria had comparable diagnostic performance with the Sokolow–Lyon criteria with 14.8% (10.4% to 20.1%) sensitivity and 96.7% (94.9% to 97.8%) specificity for detecting LVH. Again, a fall in sensitivity was seen with obesity from 21.1% (11.1% to 36.4%) to 11.9% (6.2% to 21.8%) with no change in specificity (table 2).

### Defining the effect of obesity on LV anatomical axis

When considering the whole study cohort, the incidence of a more leftward anatomical axis (defined as the lowest coronal axis tertile) increased from 13% in normal weight to 58% in the obesity, with leftward displacement of the LV axis in the coronal plane and superior displacement in the sagittal plane by on average 17° (both p<0.001).

### The effect of anatomical axis deviation on ECG voltage criteria

In order to determine the effect of an increasing leftward anatomical axis on ECG voltage criteria, subjects were matched for both LV mass and BMI. This showed that, for a given LV mass and BMI, a leftward deviation of the LV anatomical axis was associated with a significant decrease in Sokolow–Lyon amplitude (by 3–5 mm, p<0.05 all analyses). Similar results were seen using ANCOVA analysis investigating the effects of LV axis deviation on ECG criteria (covariates in model evaluated at LV mass 133 g, BMI 28.2 kg/m^2^ (mean values for the cohort)) with reducing Sokolow–Lyon voltage with increasing lateral axis displacement (lowest tertile 22.6 mm, middle tertile 21.5 mm, obese 18.9 mm, all post hoc comparisons p<0.05). There were no significant changes in Cornell criteria (p>0.99 all analyses).

### The effect of obesity on ECG voltage criteria

In order to determine the effect of obesity per se, subjects were matched for both LV mass and LV anatomical axis. This showed that, for any given LV mass and anatomical orientation, increasing BMI is associated with a significant decrease in Sokolow–Lyon index (overweight by 2–4 mm, obese by 3–5 mm, p<0.05 all analyses). Similar results were seen using ANCOVA analysis investigating the effects of obesity on ECG criteria (covariates in model evaluated at LV mass 133 g, LV coronal axis 37° (mean values for the cohort)) with reducing Sokolow–Lyon index with increasing BMI (normal 23.0 mm, overweight 20.6 mm, obese 19.5 mm, all post hoc comparisons p<0.05).

### Defining a BMI adjustment factor for the Sokolow–Lyon index

The adjustment factors for the overweight and obese cohorts were calculated as the maximum sum of the effects of both BMI and leftward anatomical LV axis deviation. This showed that when matched for LV mass, on average, a 4 mm reduction in ECG voltage amplitude was observed in overweight and an 8 mm reduction was observed with obesity. As no significant change in Cornell criteria was observed with increasing BMI, no adjustment factor was calculated.

### The effect of adjusting ECG criteria on diagnostic performance in the initial cohort

When adjusting the Sokolow–Lyon index (overweight+4 mm, obesity+8 mm) for the entire initial study group, the sensitivity increased from 14.7% (10.0% to 20.8%) to 31.4% (24.9% to 38.7%) (p<0.001, table 2). The largest gain in sensitivity was observed in the obese group from 3.1.% (0.4% to 10.8%) to 27.2% (18.0% to 39.0%), with lower, but substantial gains observed in the overweight group from 12.8% to 30.8% (table 2 all p<0.001). Notably, specificity remained high at >96.9%, even after adjustment. This would suggest this simple adjustment improves the diagnostic accuracy of the Sokolow–Lyon criteria nearing that seen in normal weight subjects. In support of this, increases in Youden index (0.28 (CI 0.21 to 0.35)), positive predictive value (0.66 (CI 0.54 to 0.76)) and negative predictive value (0.84 (CI 0.81 to 0.86)) were seen (table 2).

### Validating the proposed adjustment

In order to validate the proposed ECG adjustment, this was repeated in an independent cohort of 520 participants. Of these, 48 had CMR-defined LVH (9.3%). As with the initial cohort, despite an increase in LV mass accompanying obesity (21 g, p<0.001), ECG voltage criteria decreased by 4 mm (p<0.001, table 1).

When adjusting the Sokolow–Lyon index (overweight+4 mm, obesity+8 mm) for the entire validation cohort, sensitivity increased significantly from 17·9 (8·9–28·7) to 28·1 (17·6–40·8), p<0.001, table 2. As with the initial cohort, large gains in sensitivity were observed in the obesity group, from 9·4 (1·1–28·0) to 25·0 (11.5–43·4), and also observed in the overweight group, from 8·3 (1.1–28·0) to 39·1 (19.7–61·5), table 2, all p<0.001. Again, specificity remained high even after adjustment (>93.1%). This confirms, in a validation cohort, that this simple adjustment can substantially improve the diagnostic accuracy of the Sokolow–Lyon criteria in detecting LVH (Youden index increases from 0.12 (CI 0.02 to 0.23) to 0.30 (CI 0.17 to 0.42), ROC AUC increases from 0.60 to 0.70). However, due to the small sample size of non-Europeans in this study, this adjustment has not been sufficiently validated in the non-Caucasian population.

## Discussion

Although the Sokolow–Lyon and Cornell criteria are frequently used in clinical practice and appear widely in international guidelines, in the modern era of increasing obesity, their diagnostic accuracy is well below an acceptable level for a diagnostic screening tool. This study has shown that by incorporating BMI into the ECG algorithm by a simple adjustment, the diagnostic sensitivity can be improved without a significant decrease in specificity.

### The challenges of ECG screening for LVH in the modern population

In current practice, LVH is most accurately determined by CMR, as its accuracy far exceeds that of either echocardiography or ECG.^22^ However, the greater availability, simplicity of operation and lower cost associated with the ECG have resulted in its continued worldwide use. However, obesity affects the surface ECG significantly, reducing voltage amplitude through a combination of leftward LV axis deviation, increased chest wall fat and increased pericardial fat. In this study, obesity was observed to reduce the sum of the R wave amplitude in V5 or 6 and the S wave in V1 by up to 8 mm. Indeed, we demonstrate that the sensitivity of the Sokolow–Lyon criteria is only 3.1% in obesity, with a specificity reaching 99.0%. Although the specificity seems excellent, this likely reflects the fact that in obesity the degree of LVH required to generate >35 mm Sokolow–Lyon index is much greater, reducing its diagnostic power (reflected by the Youden index of 0.11). It is quite clear from this study that the Sokolow–Lyon criteria are completely inadequate to be used as a diagnostic screening test in the modern era of obesity. Although the Cornell criteria are seen here to be less vulnerable to increasing BMI, they also have poor diagnostic sensitivity (14·8% and 11·9%, respectively).

### Adjusting the ECG for obesity

It has previously been shown that the diagnostic sensitivity of the ECG can be improved by accounting for obesity. However, prior studies have either used 2D echocardiography to determine LV mass, which itself is limited in obesity^10^ ^13^ ^23^ or have used complex adjustment equations based on regression, which are unsuited to time-limited modern clinical medicine.^24^ ^25^ This is the first study to use CMR to investigate the effects of both obesity and associated leftward LV axis deviation on ECG LVH criteria. We have shown that being overweight reduces the Sokolow–Lyon voltage by on average 4 mm and obese by 8 mm. When using a correction factor of +4 mm in overweight and +8 mm in obesity, the diagnostic sensitivity of this criteria is increased (by up to 30% in overweight) and to a level that approaches that seen in normal weight. Importantly, although specificity for LVH does decrease after adjustment (by up to 5.5%), it remains excellent (92.9–97.9%).

Given the global utilisation of this criteria as well as the worldwide increase in obesity, this finding is of significant clinical impact and should allow a substantial increase in the detection of anatomical LVH using ECG screening. As ECG-determined LVH appears in both European and US guidelines^4^ and is known to predict mortality,^2^ ^26^ improving ECG diagnostic performance should quickly translate into significant patient benefit. However, despite these significant improvements, the sensitivity of the ECG remains poor at around 30%. In the current era, this is a level that would preclude the ECG being taken up as a screening tool for LVH if presented as a novel diagnostic test.

## Conclusion

ECG criteria for LVH severely underestimate the prevalence of anatomical LVH, especially in the setting of obesity. We propose a simple adjustment of the ECG Sokolow–Lyon criteria (+4 mm in overweight, +8 mm in obesity) that improves the diagnostic sensitivity for the detection of anatomical LVH without significantly decreasing the diagnostic specificity.

**Key messages**

**What is already known on this subject?**

- The electrocardiogram (ECG) remains the most widely used clinical screening tool for the detection of left ventricular hypertrophy (LVH), and yet existing diagnostic criteria are increasingly insensitive due to their inaccuracy in higher body weights.

**What might this study add?**

- Adjusting the Sokolow–Lyon index by +4 mm in patients with overweight (body mass index (BMI) is 25–30 kg/m^2^) and by +8 mm in patients with obesity (BMI >30 kg/m^2^) improves the diagnostic accuracy for detecting LVH.

**How might this impact on clinical practice?**

- This simple, clinically usable adjustment criterion significantly improves the power of the 12-lead ECG to detect LVH. Improving the diagnostic accuracy of the ECG should increase the number of patients identified with asymptomatic end organ damage in at-risk populations, improve risk stratification and may reduce the need for further unnecessary investigation.
